# Factors associated with adherence to self-isolation and lockdown measures in the UK: a cross-sectional survey

**DOI:** 10.1016/j.puhe.2020.07.024

**Published:** 2020-10

**Authors:** L.E. Smith, R. Amlȏt, H. Lambert, I. Oliver, C. Robin, L. Yardley, G.J. Rubin

**Affiliations:** a King's College London, Institute of Psychiatry, Psychology and Neuroscience, UK; b Public Health England, Behavioural Science Team, Emergency Response Department Science and Technology, UK; c University of Bristol, Department of Population Health Sciences, Bristol Medical School, UK; d Public Health England, Field Epidemiology, Field Service, National Infection Service, Bristol, UK; e Public Health England, Field Epidemiology, Field Service, National Infection Service, Liverpool, UK; f University of Bristol, School of Psychological Sciences, UK

**Keywords:** COVID-19, Behaviour, Social distancing, Physical distancing, Government measures, Adherence

## Abstract

**Objectives:**

To investigate factors associated with adherence to self-isolation and lockdown measures due to COVID-19 in the UK.

**Study design:**

Online cross-sectional survey.

**Methods:**

Data were collected between 6th and 7th May 2020. A total of 2240 participants living in the UK aged 18 years or older were recruited from YouGov's online research panel.

**Results:**

A total of 217 people (9.7%) reported that they or someone in their household had symptoms of COVID-19 (cough or high temperature/fever) in the last 7 days. Of these people, 75.1% had left the home in the last 24 h (defined as non-adherent). Men were more likely to be non-adherent, as were people who were less worried about COVID-19, and who perceived a smaller risk of catching COVID-19. Adherence was associated with having received help from someone outside your household. Results should be taken with caution as there was no evidence for associations when controlling for multiple analyses. Of people reporting no symptoms in the household, 24.5% had gone out shopping for non-essentials in the last week (defined as non-adherent). Factors associated with non-adherence and with a higher total number of outings in the last week included decreased perceived effectiveness of government ‘lockdown’ measures, decreased perceived severity of COVID-19 and decreased estimates of how many other people were following lockdown rules. Having received help was associated with better adherence.

**Conclusions:**

Adherence to self-isolation is poor. As we move into a new phase of contact tracing and self-isolation, it is essential that adherence is improved. Communications should aim to increase knowledge about actions to take when symptomatic or if you have been in contact with a possible COVID-19 case. They should also emphasise the risk of catching and spreading COVID-19 when out and about and the effectiveness of preventative measures. Using volunteer networks effectively to support people in isolation may promote adherence.

## Highlights

• 75% of those with household COVID-19 symptoms had left home in the last 24 h.
• Total outings and shopping for non-essentials indicated non-adherence to lockdown.
• Decreased perceived effectiveness of lockdown measures was linked to non-adherence.
• Non-adherence was also linked with decreased perceived severity of COVID-19.
• Perceived social norms were associated with adherence to lockdown measures.

## Introduction

During the coronavirus pandemic, governments have imposed restrictions of movement to prevent the spread of the virus. Commonly used measures are self-isolation, in which people who are ill separate themselves from others, and quarantine, in which people who may have been exposed to the illness separate themselves from others.^1^ On 23rd March 2020, the UK government introduced ‘lockdown’ measures to slow the spread of COVID-19.^2^^,^^3^ These required people to: stay at home except for several, limited reasons; not leave the home at all for 7 days, if suffering from a new continuous cough or fever; and not leave the home at all for 14 days, if someone else in the household developed cough or fever.

Adherence to these measures may be influenced by multiple factors. According to Protection Motivation Theory,^4^ uptake of a protective behaviour is influenced by your appraisal of a threat, including its severity and your susceptibility to it, and your appraisal of the behaviour, including perceptions about its efficacy, your ability to perform it and the costs associated with it. A review of quarantine measures in previous public health crises found that knowledge and perceived social norms were also associated with adherence to quarantine.^5^ Conversely, fear of missing out, perceived social pressure, perceived legal consequences, running out of supplies (e.g. food or medicine) and financial pressures were associated with decreased adherence. There is some evidence that people who think they have had COVID-19 are less likely to adhere to lockdown measures.^6^

In this study, we investigated factors associated with adherence to lockdown measures in a demographically representative sample of the UK adult population.

## Method

### Design

We commissioned the market research company YouGov to carry out this cross-sectional survey, between 6th and 7th May 2020.

### Participants

Participants (*n* = 2240) were recruited from YouGov's online research panel (*n* = 800,000+ UK adults) and were eligible if they aged 18 years or older and living in the UK. Quota sampling was used, based on age, gender, social grade, level of education and Government Office Region, to ensure that the sample was broadly representative of the UK general population. In total, 74 participants were excluded because of a lack of data for sociodemographic variables, suspiciously fast completion of the survey or providing identical answers to multiple consecutive questions. Participants were reimbursed in points equivalent to approximately 50p.

### Study materials

Full survey materials are available in the Supplementary Materials.

#### Outcome measures

We asked participants how many times they had left their home ‘in the past 24 h’ and ‘in the past seven days’: to go to the shops for groceries, toiletries or medicine; to go to the shops for other items; for exercise; for a medical purpose excluding going to the shops/pharmacy for medicine; to go to work; to help someone else; and to meet friends or family who they did not live with.

#### Psychological and situational factors

We asked participants if they or a household member had experienced any of 13 symptoms, including cough and high temperature/fever, in the past 7 and 14 days, respectively.

We asked participants whether they thought they had ‘had, or currently have, coronavirus’ and if they were currently self-isolating.

We asked participants a series of true/false statements about the current UK government guidance.

We asked participants how worried they were about COVID-19 on a five-point Likert-type scale from ‘not at all worried’ to ‘extremely worried’.

To measure perceived social norms, we asked participants to estimate the percentage of people the same age as them who were fully following the UK government's recommendations to stay at home.

We asked participants whether they thought the current lockdown had made their physical health better or worse. Possible answers were ‘a lot better’, ‘a little better’, ‘no difference’, ‘a little worse’ and ‘a lot worse’.

We asked participants to rate their general health on a five-point Likert-type scale from ‘poor’ to ‘excellent’ using one item from the SF-36.^7^

We asked participants if they had helped someone, or received help from someone, outside their household in the past 7 days (yes/no).

We asked participants to rate 14 perception statements on a five-point Likert scale from ‘strongly disagree’ to ‘strongly agree’. Statements included the perceived severity of COVID-19, perceived effectiveness of government measures, perceived likelihood of catching and spreading COVID-19, perceived costs of following government measures, fear of losing touch with friends and relatives, social pressure from friends and family to follow government measures, perceived legal consequences of not following government measures and positive consequences of the lockdown.

#### Personal and clinical characteristics

We asked participants to report their age, gender, employment status, highest educational or professional qualification and marital status. We also asked whether there was a child in their household, whether they or someone else in their household received a letter from the National Health Service telling them they were extremely clinically vulnerable to COVID-19, and whether they lived alone. Participants were asked for their postcode to determine indices of multiple deprivation (IMD) and whether they lived in an urban or rural area. We also collected social grade.

We asked participants if their primary home had access to any outdoor space, and whether they were pet owners.

### Ethics

Ethical approval for this study was granted by the King's College London Research Ethics Committee (reference: LRS-19/20-18687).

### Power

We calculated achieved power for the analyses (in households with and without symptoms) using *post-hoc* power calculations. Achieved power is presented underneath relevant analyses.

### Analysis

For all variables, unless stated otherwise, we coded answers of ‘don't know’ as missing data.

We investigated whether out-of-home activity (total number of outings, percentage of people reporting shopping for non-essentials, going to meet friends or family, and having visitors to their home) differed by presence of symptoms in the household.

We split the sample by presence of symptoms in the household. Among those who reported symptoms in their household in the last 7 days, we defined those who reported having gone out in the last 24 h as not adhering to self-isolation measures. We ran a series of logistic regressions investigating univariable associations between personal and clinical factors, psychological and situational factors, and having left the home in the past 24 h. We ran a second set of logistic regressions controlling for personal and clinical characteristics.

Among those who reported no symptoms in the household, we used UK government guidelines that were in force at the time of data collection^2^ to define non-adherence (shopping for non-essentials, meeting friends or family and having visitors to your home). We ran a series of linear regressions investigating univariable associations between personal and clinical factors, psychological and situational factors, and total number of outings reported in the past 7 days. We ran a second set of linear regressions controlling for personal and clinical characteristics (personal and clinical characteristics entered as the first block, other independent variables as the second block). We ran a series of logistic regressions investigating univariable associations between personal and clinical factors, psychological and situational factors, and going out shopping for items other than groceries, toiletries or medicines (non-essentials) in the past 7 days. We ran a second set of logistic regressions controlling for personal and clinical characteristics.

Weighting data by age, gender, social grade, highest level of education and region altered prevalence of outcome behaviours only slightly. We therefore used unweighted data in our analyses.

#### Sensitivity analyses

Owing to the large number of analyses (*n* = 39) run on each outcome, we applied a Bonferroni correction to our results (*P* ≤ 0.001). Those meeting this criterion are marked by a double asterisk (∗∗) in the tables.

## Results

*Results of adjusted analyses are reported narratively; unadjusted results are reported in tables*.

A minority of participants (9.7%, *n* = 217) reported that either they or a household member had a cough or a high temperature/fever in the last 7 or 14 days, respectively. Participants' characteristics are shown in Table 1. Male participants were more likely to report symptoms in their household. There were no other differences between groups.

**Table 1:** Participants’ personal and clinical characteristics, by report of symptoms in the household.

Participants' characteristics	Level	No symptoms in household; *n* = 1945	Symptoms in household; *n* = 217	*P*-value
Gender	Male	915 (47.0)	124 (57.1)	0.01∗
	Female	1030 (53.0)	93 (42.9)	
Age, years	18–24	76 (3.9)	10 (4.6)	0.46
	25–34	259 (13.3)	35 (16.1)	
	35–44	347 (17.8)	37 (17.1)	
	45–54	363 (18.7)	31 (14.3)	
	55 and older	900 (46.3)	104 (47.9)	
Child in the household	None	1428 (74.3)	153 (72.9)	0.65
	Child present	494 (25.7)	57 (27.1)	
Clinically extremely vulnerable (self)	No	1760 (93.1)	190 (90.9)	0.25
	Yes	131 (6.9)	19 (9.1)	
Employment status	Not working	903 (46.6)	102 (47.0)	0.87
	Working	1042 (53.6)	115 (53.0)	
Highest educational or professional qualification	GCSE/vocational/A-level/No formal qualifications	856 (44.9)	86 (40.4)	0.21
	Degree or higher (Bachelors, Masters, PhD)	1052 (55.1)	127 (59.6)	
IMD	More deprived area	851 (43.8)	103 (47.5)	0.30
	Less deprived area	1094 (56.2)	114 (52.5)	
Social grade	ABC1	1184 (60.9)	133 (61.3)	0.91
	C2DE	761 (39.1)	84 (38.7)	
Urban/rural	Urban	1462 (77.2)	166 (79.4)	0.46
	Rural	433 (22.8)	43 (20.6)	
Living alone	Yes	402 (20.7)	34 (15.8)	0.09
	No	1543 (79.3)	181 (84.2)	
Marital status	Married/civil partnership/living as married	1233 (63.8)	142 (65.4)	0.62
	Separated/divorced/widowed/never married	701 (36.2)	75 (34.6)	

*∗P* ≤ 0.05.

### Symptoms in household

Of participants who reported symptoms in their household (*n* = 217), 75.1% (*n* = 163, 95% confidence interval [CI; 69.3–80.9]) reported leaving the home at least once in the past 24 h. This finding has been reported elsewhere.^8^

A few participants (*n* = 54, 2.4%) reported going out many times; we grouped responses of over 20 times in the past 7 days. There was no difference in out-of-home activity by presence of symptoms in the household (total number of outings made in the last week, *t*(2160) = 0.20, *P* = .84; percentage of people reporting shopping for non-essentials, χ^2^ (1, 2162) = 0.38, *P* = .54; having had a visitor to one's home, χ^2^ (1, 2076) = 0.40, *P* = .53; or going to meet friends or family, χ^2^ (1, 2162) = 1.34, *P* = .25).

Of those who reported symptoms in the household, 34.1% (*n* = 74) reported that they were self-isolating. Of those ‘self-isolating’, 60.8% (*n* = 45) nonetheless reported having gone out in the last 24 h.

Men were more likely to leave the home in the last 24 h (see Table 2).

**Table 2:** Associations between personal and clinical characteristics of participants who reported symptoms in their household in the last week and having left the home in the past 24 hours.

Participants' characteristics	Level	Did not go out in the past 24 h; *n* = 54	Went out in the past 24 h; *n* = 163	Odds ratio (95% CI)	Adjusted odds ratio (95% CI)^a^
Gender	Male	23 (18.5)	101 (81.5)	Reference	Reference
	Female	31 (33.3)	62 (66.7)	0.46 (0.24–0.85)∗	0.32 (0.14–0.76)∗
Age, years	18–24	4 (40.0)	6 (60.0)	Reference	Reference
	25–34	8 (22.9)	27 (77.1)	2.25 (0.51–9.99)	2.37 (0.29–19.26)
	35–44	6 (16.2)	31 (83.8)	3.44 (0.74–16.03)	2.58 (0.31–21.54)
	45–54	10 (32.3)	21 (67.7)	1.40 (0.32–6.10)	1.22 (0.17–8.81)
	55 and older	54 (24.9)	163 (75.1)	2 (0.52–7.64)	2.40 (0.33–17.55)
Child in the household	None	44 (28.8)	109 (71.2)	Reference	Reference
	Child present	7 (12.3)	50 (87.7)	2.88 (1.21–6.85)∗	2.88 (0.90–9.21)
Clinically extremely vulnerable (self)	No	44 (23.2)	146 (76.8)	Reference	Reference
	Yes	8 (42.1)	11 (57.9)	0.41 (0.16–1.09)	0.38 (0.10–1.48)
Employment status	Not working	34 (33.3)	68 (66.7)	Reference	Reference
	Working	20 (17.4)	95 (82.6)	2.37 (1.26–4.48)∗	2.51 (0.92–6.83)
Highest educational or professional qualification	GCSE/vocational/A-level/No formal qualifications	22 (25.6)	64 (74.4)	Reference	Reference
	Degree or higher (Bachelors, Masters, PhD)	29 (22.8)	98 (77.2)	1.16 (0.61–2.20)	0.70 (0.28–1.76)
IMD	More deprived area	25 (24.3)	78 (75.7)	Reference	Reference
	Less deprived area	29 (25.4)	85 (74.6)	0.94 (0.51–1.74)	1.91 (0.73–5.02)
Social grade	ABC1	37 (27.8)	96 (72.2)	Reference	Reference
	C2DE	17 (20.2)	67 (79.8)	1.52 (0.79–2.92)	2.39 (0.89–6.39)
Urban/rural	Urban	39 (23.5)	127 (76.5)	Reference	Reference
	Rural	12 (27.9)	31 (72.1)	0.79 (0.37–1.69)	0.62 (0.22–1.78)
Living alone	Yes	11 (32.4)	23 (67.6)	Reference	Reference
	No	41 (22.7)	140 (77.3)	1.63 (0.73–3.63)	0.52 (0.12–2.15)
Marital status	Married/civil partnership/living as married	27 (19.0)	115 (81.0)	Reference	Reference
	Separated/divorced/widowed/never married	27 (36.0)	48 (64.0)	0.42 (0.22–0.78)∗	0.42 (0.12–1.46)
Clinically extremely vulnerable (household member)^b^	No	38 (23.6)	123 (76.4)	Reference	Reference
	Yes	3 (21.4)	11 (78.6)	1.13 (0.30–4.27)	3.47 (0.47–25.46)
Home includes access to outside space	No	4 (36.4)	7 (63.6)	Reference	Reference
	Yes	50 (24.3)	156 (75.7)	1.78 (0.50–6.34)	0.36 (0.03–4.00)
Pet ownership	No	31 (31.6)	67 (68.4)	Reference	Reference
	Yes	23 (19.3)	96 (80.7)	1.93 (1.04–3.60)∗	1.72 (0.72–4.11)

∗*P* ≤ 0.05.

a Adjusting for gender, age, having a child in the household, being extremely clinically vulnerable oneself, employment status, highest level of education or professional qualification, indices of multiple deprivation, social grade, living in a rural or urban area, living alone, marital status and region.

b Adjusted analyses for this variable did not control for living alone, as by definition all participants asked this question lived in a household with someone else.

Non-adherence to self-isolation (reporting having left home in the last 24 h) was associated with: thinking that the lockdown had made your mental health worse; feeling a greater sense of community with your neighbourhood due to COVID-19 (see Table 3). Adherence to self-isolation (reporting not having left home in the last 24 h) was associated with: reporting that you were self-isolating; increased worry about COVID-19; having received help from someone outside your household in the last seven days because of COVID-19; and increased perceived likelihood of catching COVID-19.

**Table 3:** Associations between psychological and situational factors and having left the home in the past 24 hours in participants who reported symptoms in the household.

Participants' characteristics	Level	Did not go out in the past 24 h; *n* = 54	Went out in the past 24 h; *n* = 163	Odds ratio (95% CI)	Adjusted odds ratio (95% CI)^a^
Had, or currently have, COVID-19	Think have not had COVID-19 and do not have it now	27 (20.8)	103 (79.2)	Reference	Reference
	Think have had COVID-19 or have it now	17 (37.0)	29 (63.0)	0.45 (0.21–0.93)∗	0.32 (0.09–1.17)
Self-isolating	Not self-isolating	25 (17.5)	118 (82.5)	Reference	Reference
	Self-isolating	29 (39.2)	45 (60.8)	0.33 (0.17–0.62)∗∗	0.23 (0.09–0.61)∗
Understanding of government measures if no-one in household was symptomatic	Incorrect/unsure	34 (26.6)	94 (73.4)	Reference	Reference
	Correct	20 (22.5)	69 (77.5)	1.25 (0.66–2.35)	0.95 (0.40–2.23)
Understanding of government measures if someone in household was symptomatic	Incorrect/unsure	49 (24.1)	154 (75.9)	Reference	Reference
	Correct	5 (35.7)	9 (64.3)	0.57 (0.18–1.79)	1.31 (0.29–5.96)
Worry about COVID-19	5-point scale, 1 = not at all worried to 5 = extremely worried	N = 54, M = 3.70, SD = 0.92	N = 163, M = 3.44, SD = 1.02	0.77 (0.56–1.05)	0.61 (0.37–0.98)∗
Perceived social norms	Percentage (range 0–100)	N = 46, M = 72.13, SD = 20.53	N = 151, M = 69.84, SD = 17.39	0.99 (0.97–1.01)	0.99 (0.97–1.02)
Perceptions about impact on mental health	5-point scale, 1 = a lot better to 5 = a lot worse	N = 54, M = 3.37, SD = 1.07	N = 160, M = 3.57, SD = 0.96	1.22 (0.90–1.67)	1.61 (1.03–2.500)∗
Perceptions about impact on physical health	5-point scale, 1 = a lot better to 5 = a lot worse	N = 54, M = 3.54, SD = 0.91	N = 162, M = 3.38, SD = 0.91	0.82 (0.58–1.16)	0.77 (0.48–1.25)
Self-reported general health	5-point scale, 1 = poor to 5 = excellent	N = 54, M = 2.33, SD = 1.13	N = 161, M = 2.75, SD = 0.96	1.51 (1.10–2.06)∗	1.53 (0.99–2.38)
Helped someone outside household	No	44 (28.6)	110 (71.4)	Reference	Reference
	Yes	9 (14.8)	52 (85.2)	2.31 (1.05–5.09)∗	2.38 (0.86–6.61)
Received help from someone outside household	No	37 (20.8)	141 (79.2)	Reference	Reference
	Yes	16 (43.2)	21 (56.8)	0.34 (0.16–0.73)∗	0.30 (0.09–0.96)∗
If I completely follow the government's advice, I will lose touch with my friends and relatives	5-point scale, 1 = strongly disagree to 5 = strongly agree	N = 53, M = 1.96, SD = 1.16	N = 161, M = 2.25, SD = 1.26	1.23 (0.94–1.61)	1.20 (0.82–1.76)
My friends or family will disapprove if I don't follow the government's advice	5-point scale, 1 = strongly disagree to 5 = strongly agree	N = 52, M = 3.92, SD = 1.19	N = 159, M = 4.05, SD = 0.90	1.14 (0.83–1.56)	1.17 (0.76–1.80)
If I don't follow the government's advice, I could get in trouble with the police	5-point scale, 1 = strongly disagree to 5 = strongly agree	N = 53, M = 3.98, SD = 0.84	N = 159, M = 3.83, SD = 0.93	0.83 (0.58–1.18)	0.89 (0.56–1.39)
If I follow the government's advice, it will help save lives	5-point scale, 1 = strongly disagree to 5 = strongly agree	N = 54, M = 4.54, SD = 0.88	N = 161, M = 4.39, SD = 0.89	0.81 (0.55–1.19)	0.73 (0.43–1.23)
If I follow the government's advice, it will help protect the NHS	5-point scale, 1 = strongly disagree to 5 = strongly agree	N = 54, M = 4.57, SD = 0.66	N = 161, M = 4.47, SD = 0.81	0.82 (0.53–1.27)	0.90 (0.51–1.57)
If I catch coronavirus, I may become very ill	5-point scale, 1 = strongly disagree to 5 = strongly agree	N = 53, M = 4.51, SD = 0.72	N = 156, M = 4.45, SD = 0.90	0.92 (0.63–1.34)	1.06 (0.64–1.74)
If I catch coronavirus, it will have a severe impact on my family's well-being	5-point scale, 1 = strongly disagree to 5 = strongly agree	N = 52, M = 4.15, SD = 1.04	N = 158, M = 4.18, SD = 1.04	1.03 (0.76–1.39)	1.34 (0.87–2.08)
If I leave home and meet other people, I could pass coronavirus to someone else	5-point scale, 1 = strongly disagree to 5 = strongly agree	N = 53, M = 4.62, SD = 0.56	N = 157, M = 4.52, SD = 0.75	0.79 (0.49–1.28)	0.61 (0.29–1.27)
If I leave home and meet other people, I could catch coronavirus	5-point scale, 1 = strongly disagree to 5 = strongly agree	N = 54, M = 4.74, SD = 0.48	N = 161, M = 4.5, SD = 0.73	0.51 (0.29–0.92)∗	0.40 (0.16–0.99)∗
If I follow the government's advice, it will have a negative impact on how much money I have	5-point scale, 1 = strongly disagree to 5 = strongly agree	N = 53, M = 2.55, SD = 1.29	N = 159, M = 2.71, SD = 1.28	1.11 (0.87–1.41)	1.16 (0.85–1.60)
Because of the current lockdown, there is more conflict between people that I live with	5-point scale, 1 = strongly disagree to 5 = strongly agree	N = 52, M = 2.15, SD = 1.13	N = 160, M = 2.28, SD = 1.27	1.08 (0.84–1.40)	1.26 (0.85–1.85)
If I follow the government's advice, I will not be able to carry out important religious activities	5-point scale, 1 = strongly disagree to 5 = strongly agree	N = 50, M = 2.58, SD = 1.49	N = 148, M = 2.69, SD = 1.31	1.06 (0.84–1.35)	1.08 (0.77–1.49)
I am enjoying spending more time at home during the lockdown	5-point scale, 1 = strongly disagree to 5 = strongly agree	N = 54, M = 3.46, SD = 1.21	N = 162, M = 3.19, SD = 1.22	0.82 (0.64–1.07)	0.83 (0.59–1.18)
Because of coronavirus, I feel a sense of community with other people in my neighbourhood	5-point scale, 1 = strongly disagree to 5 = strongly agree	N = 54, M = 2.98, SD = 1.22	N = 162, M = 3.25, SD = 1.14	1.22 (0.94–1.60)	1.52 (1.03–2.24)∗

∗*P* ≤ 0.05.

∗∗*P* ≤ 0.001.

a Adjusting for gender, age, having a child in the household, being extremely clinically vulnerable oneself, employment status, highest level of education or professional qualification, indices of multiple deprivation, social grade, living in a rural or urban area, living alone, marital status and region.

#### Power

For analyses where symptoms were present in the household, we achieved 94% power to detect small effect sizes in logistic regression analyses (odds ratio [OR] = 1.68,^9^ α = .05, sample size *n* = 217, probability of having left the home = 0.75, one-tailed logistic regression; 89% power when using a two-tailed logistic regression).^10^

### No symptoms in household

Of those who reported no symptoms in their household, 24.5% reported having gone out to shop for items other than groceries, toiletries or medicines (*n* = 476, 95% CI [22.6–26.4]), 5.9% reported meeting up with friends and/or family that they did not live with (*n* = 114, 95% CI [4.8–6.9]), and 4.3% reported having had visitors to their home in the last 7 days (*n* = 81, 95% CI [3.4–5.3]). The mean number of outings made by participants was 6.77 (standard deviation [SD] = 5.07, median = 6, mode = 0).

Personal and clinical factors (gender, age, having a child in the household, being extremely clinically vulnerable oneself, employment status, highest level of education or professional qualification, IMD, social grade, living in a rural or urban area, living alone, marital status and region [results for region not reported]) explained 8.0% of the variance in number of outings in the past week (see Table 4). More outings were made by men, those who reported working and who lived in rural areas. Fewer outings were made by those who were clinically extremely vulnerable and who lived in more deprived areas. Having a pet was also associated with going out more often.

**Table 4:** Associations between personal and clinical characteristics and total number of outings in the past week in participants who reported no symptoms in the household.

Participants' characteristics	Level	Number of outings	Total number of outings in the past week									
			Unadjusted analyses					Adjusted analyses^a^				
			Model			Regression coefficient		Model			Regression coefficient	
			*F*	Adjusted *R*^2^	*P*-value	Β	*P*-value	*F*	Adjusted *R*^2^	*P*-value	β	*P*-value
Gender	Male, *n* = 915	M = 7.22, SD = 5.27										
	Female, *n* = 1030	M = 6.37, SD = 4.85	13.89	0.007	<0.001∗∗	−0.08	<0.001∗∗				−0.09	<0.001∗∗
Age, years	18–24, *n* = 76	M = 5.04, SD = 5.26										
	25–34, *n* = 259	M = 7.50, SD = 5.05										
	35–44, *n* = 347	M = 7.35, SD = 4.68										
	45–54, *n* = 363	M = 7.63, SD = 5.22										
	55 and older, *n* = 900	M = 6.13, SD = 5.04	7.61	0.003	0.01∗	−0.06	.01∗				0.00	0.91
Child in the household	None, *n* = 1428	M = 6.59, SD = 5.13										
	Child present, *n* = 494	M = 7.34, SD = 4.91	7.84	0.004	0.01∗	0.06	0.01∗				0.00	0.95
Clinically extremely vulnerable (self)	No, *n* = 1760	M = 6.97, SD = 5.00										
	Yes, *n* = 131	M = 4.39, SD = 5.27	32.22	0.017	<0.001∗∗	−0.13	<0.001∗∗				−0.10	<0.001∗∗
Employment status	Not working, *n* = 903	M = 5.48, SD = 4.72										
	Working, *n* = 1042	M = 7.88, SD = 5.11	114.62	0.055	<0.001∗∗	0.24	<0.001∗∗				0.24	<0.001∗∗
Highest educational or professional qualification	GCSE/vocational/A-level/No formal qualifications, *n* = 856	M = 6.59, SD = 5.26										
	Degree or higher (Bachelors, Masters, PhD), *n* = 1052	M = 6.96, SD = 4.93	2.47	0.001	0.12	0.04	0.12				0.01	0.56
IMD	More deprived area, *n* = 851	M = 6.35, SD = 5.17	10.22	0.005	0.001∗∗	0.07	0.001∗∗				0.06	0.007∗
	Less deprived area, *n* = 1094	M = 7.09, SD = 4.97										
Social grade	ABC1, *n* = 1184	M = 6.94, SD = 4.84										
	C2DE, *n* = 761	M = 6.50, SD = 5.40	3.45	0.001	0.06	−0.04	0.06				0.03	0.29
Urban/rural	Urban, *n* = 1462	M = 6.62, SD = 5.08										
	Rural, *n* = 433	M = 7.01, SD = 4.97	2.04	0.001	0.15	0.03	0.15				0.05	0.04∗
Living alone	Yes, *n* = 402	M = 6.34, SD = 5.19										
	No, *n* = 1543	M = 6.88, SD = 5.04	3.59	0.001	0.06	0.04	0.06				0.01	0.83
Marital status	Married/civil partnership/living as married, *n* = 1233	M = 6.92, SD = 4.98										
	Separated/divorced/widowed/never married, *n* = 701	M = 6.52, SD = 5.24	2.79	0.001	0.10	−0.04	0.10				−0.01	0.81
Model			–	–	–	–	–	13.71	0.079	<0.001∗∗		
Clinically extremely vulnerable (household member)^b^	No, *n* = 1374	M = 6.92, SD = 4.95										
	Yes, *n* = 125	M = 6.59, SD = 5.65	0.50	0.000	0.48	−0.02	0.48	9.86	0.070	<0.001∗∗	−0.01	0.60
Home includes access to outside space	No, *n* = 146	M = 6.60, SD = 5.16										
	Yes, *n* = 1799	M = 6.78, SD = 5.07	0.19	0.000	0.67	0.01	0.67	12.65	0.078	<0.001∗∗	0.00	0.88
Pet ownership	No, *n* = 1072	M = 6.19, SD = 4.80										
	Yes, n = 873	M = 7.48, SD = 5.30	31.13	0.015	<0.001∗∗	0.13	<0.001∗∗	14.56	0.090	<0.001∗∗	0.11	<0.001∗∗

∗*P* ≤ 0.05.

∗∗*P* ≤ 0.001.

a Adjusting for gender, age, having a child in the household, being extremely clinically vulnerable oneself, employment status, highest level of education or professional qualification, indices of multiple deprivation, social grade, living in a rural or urban area, living alone, marital status and region. Personal and clinical characteristics entered as first block, other independent variables entered as second block.

b Adjusted analyses for this variable did not control for living alone, as by definition all participants asked this question lived in a household with someone else.

More outings in the past week were associated with: helping someone outside your household; decreased perceived effectiveness of government measures; thinking that you would lose touch with friends and relatives if you followed government advice; not enjoying spending more time at home during the lockdown; better self-reported general health; decreased perceived severity of COVID-19; decreased perceived likelihood of spreading COVID-19; decreased perceived legal consequences of not following government advice; decreased perceived social pressure from friends and family to follow government measures; full, correct knowledge of government measures if no-one in the household was symptomatic; believing that you have had or currently have COVID-19; increased perceived financial cost of following government measures; and decreased perceived social norms (see Table 5). Fewer outings were associated with: receiving help from someone outside your household; decreased perceived impact of lockdown on physical health; reporting that you were self-isolating; increased worry about COVID-19; and increased perceived likelihood of catching COVID-19.

**Table 5:** Associations between psychological and situational factors and total number of outings in the past week in participants who reported no symptoms in the household.

Participants' characteristics	Level	Number of outings	Total number of outings in the past week									
			Unadjusted analyses					Adjusted analyses^a^				
			Model			Regression coefficient		Model			Regression coefficient	
			*F*	Adjusted *R*^2^	*P*-value	β	*P*-value	*F*	Adjusted *R*^2^	*P*-value	β	*P*-value
Had, or currently have, COVID-19	Think have not had COVID-19 and do not have it now, *n* = 1532	M = 6.58, SD = 5.00										
	Think have had COVID-19 or have it now, *n* = 155	M = 8.00, SD = 5.34	11.21	0.006	0.001∗∗	0.08	0.001∗∗	11.90	0.084	<0.001∗∗	0.07	0.006∗∗
Self-isolating	Not self-isolating, *n* = 1491	M = 7.66, SD = 4.85										
	Self-isolating, *n* = 454	M = 3.85, SD = 4.67	174.65	0.083	<0.001∗∗	−0.29	<0.001∗∗	23.73	0.142	<0.001∗∗	−0.28	<0.001∗∗
Understanding of government measures if no-one in household was symptomatic	Incorrect/unsure, *n* = 1052	M = 6.41, SD = 5.23										
	Correct, *n* = 893	M = 7.19, SD = 4.85	11.66	0.005	0.001∗∗	0.08	0.001∗∗	13.21	0.082	<0.001∗∗	0.06	0.01∗
Understanding of government measures if someone in household was symptomatic	Incorrect/unsure, *n* = 1834	M = 6.77, SD = 5.10										
	Correct, *n* = 111	M = 6.68, SD = 4.66	0.3	0.000	0.86	0.00	0.86	12.67	0.079	<0.001∗∗	−0.01	0.59
Worry about COVID-19	5-point scale, 1 = not at all worried to 5 = extremely worried, *n* = 1938	M = 6.78, SD = 5.07	127.48	0.061	<0.001∗∗	−0.25	<0.001∗∗	20.85	0.127	<0.001∗∗	−0.23	<0.001∗∗
Perceived social norms	Percentage (range 0–100), *n* = 1742	M = 6.89, SD = 5.03	8.48	0.004	0.004∗	−0.07	0.004∗	10.79	0.073	<0.001∗∗	−0.07	0.004∗
Perceptions about impact on mental health	5-point scale, 1 = a lot better to 5 = a lot worse, *n* = 1922	M = 6.80, SD = 5.08	1.09	0.000	0.296	0.02	0.296	12.92	0.081	<0.001∗∗	0.03	0.25
Perceptions about impact on physical health	5-point scale, 1 = a lot better to 5 = a lot worse, *n* = 1927	M = 6.79, SD = 5.07	26.54	0.013	<0.001∗∗	−0.12	<0.001∗∗	14.43	0.090	<0.001∗∗	−0.10	<0.001∗∗
Self-reported general health	5-point scale, 1 = poor to 5 = excellent, *n* = 1930	M = 6.78, SD = 5.06	88.52	0.043	<0.001∗∗	0.21	<0.001∗∗	16.36	0.101	<0.001∗∗	0.16	<0.001∗∗
Helped someone outside household	No, *n* = 1469	M = 6.00, SD = 4.79										
	Yes, *n* = 459	M = 9.30, SD = 5.16	159.54	0.076	<0.001∗∗	0.28	<0.001∗∗	23.89	0.150	<0.001∗∗	0.26	<0.001∗∗
Received help from someone outside household	No, *n* = 1665	M = 7.15, SD = 5.04										
	Yes, *n* = 263	M = 4.54, SD = 4.74	61.89	0.031	<0.001∗∗	−0.18	<0.001∗∗	14.44	0.090	<0.001∗∗	−0.11	<0.001∗∗
If I completely follow the government's advice, I will lose touch with my friends and relatives	5-point scale, 1 = strongly disagree to 5 = strongly agree, *n* = 1925	M = 6.78, SD = 5.07	36.17	0.018	<0.001∗∗	−0.14	<0.001∗∗	16.48	0.102	<0.001∗∗	0.15	<0.001∗∗
My friends or family will disapprove if I don't follow the government's advice	5-point scale, 1 = strongly disagree to 5 = strongly agree, *n* = 1894	M = 6.82, SD = 5.06	26.75	0.013	<0.001∗∗	−0.12	<0.001∗∗	14.25	0.090	<0.001∗∗	−0.12	<0.001∗∗
If I don't follow the government's advice, I could get in trouble with the police	5-point scale, 1 = strongly disagree to 5 = strongly agree, *n* = 1916	M = 6.79, SD = 5.07	32.29	0.016	<0.001∗∗	−0.13	<0.001∗∗	15.22	0.095	<0.001∗∗	−0.13	<0.001∗∗
If I follow the government's advice, it will help save lives	5-point scale, 1 = strongly disagree to 5 = strongly agree, *n* = 1930	M = 6.78, SD = 5.07	51.30	0.025	<0.001∗∗	−0.16	<0.001∗∗	17.02	0.105	<0.001∗∗	−0.16	<0.001∗∗
If I follow the government's advice, it will help protect the NHS	5-point scale, 1 = strongly disagree to 5 = strongly agree, *n* = 1929	M = 6.78, SD = 5.08	30.05	0.015	<0.001∗∗	−0.12	<0.001∗∗	15.26	0.095	<0.001∗∗	−0.13	<0.001∗∗
If I catch coronavirus, I may become very ill	5-point scale, 1 = strongly disagree to 5 = strongly agree, *n* = 1917	M = 6.77, SD = 5.09	49.66	0.025	<0.001∗∗	−0.16	<0.001∗∗	15.90	0.099	<0.001∗∗	−0.15	<0.001∗∗
If I catch coronavirus, it will have a severe impact on my family's well-being	5-point scale, 1 = strongly disagree to 5 = strongly agree, *n* = 1894	M = 6.78, SD = 5.08	41.30	0.021	<0.001∗∗	−0.15	<0.001∗∗	15.21	0.096	<0.001∗∗	−0.15	<0.001∗∗
If I leave home and meet other people, I could pass coronavirus to someone else	5-point scale, 1 = strongly disagree to 5 = strongly agree, *n* = 1924	M = 6.79, SD = 5.08	24.87	0.012	<0.001∗∗	−0.11	<0.001∗∗	15.27	0.095	<0.001∗∗	−0.13	<0.001∗∗
If I leave home and meet other people, I could catch coronavirus	5-point scale, 1 = strongly disagree to 5 = strongly agree, *n* = 1929	M = 6.78, SD = 5.08	77.12	0.038	<0.001∗∗	−0.20	<0.001∗∗	18.48	0.114	<0.001∗∗	−0.19	<0.001∗∗
If I follow the government's advice, it will have a negative impact on how much money I have	5-point scale, 1 = strongly disagree to 5 = strongly agree, *n* = 1893	M = 6.81, SD = 5.09	5.22	0.002	0.02∗	0.05	0.02∗	12.37	0.078	<0.001∗∗	0.06	0.02∗∗
Because of the current lockdown, there is more conflict between people that I live with	5-point scale, 1 = strongly disagree to 5 = strongly agree, *n* = 1859	M = 6.81, SD = 5.08	2.67	0.001	0.10	0.04	0.10	12.36	0.080	<0.001∗∗	0.04	0.10
If I follow the government's advice, I will not be able to carry out important religious activities	5-point scale, 1 = strongly disagree to 5 = strongly agree, *n* = 1719	M = 6.79, SD = 5.09	1.75	0.000	0.19	0.03	0.19	11.67	0.081	<0.001∗∗	0.05	0.07
I am enjoying spending more time at home during the lockdown	5-point scale, 1 = strongly disagree to 5 = strongly agree, *n* = 1931	M = 6.76, SD = 5.07	27.82	0.014	<0.001∗∗	−0.12	<0.001∗∗	16.71	0.103	<0.001∗∗	−0.16	<0.001∗∗
Because of coronavirus, I feel a sense of community with other people in my neighbourhood	5-point scale, 1 = strongly disagree to 5 = strongly agree, *n* = 1925	M = 6.79, SD = 5.08	0.84	0.000	0.36	0.02	0.36	12.69	0.079	<0.001∗∗	0.03	0.21

∗*P* ≤ 0.05.

∗∗*P* ≤ 0.001.

a Adjusting for gender, age, having a child in the household, being extremely clinically vulnerable oneself, employment status, highest level of education or professional qualification, indices of multiple deprivation, social grade, living in a rural or urban area, living alone, marital status and region. Personal and clinical characteristics entered as first block, other independent variables entered as second block.

Going out shopping for non-essentials in the past week was associated with male participants, working and lower social grade (see Table 6).

**Table 6:** Associations between personal and clinical characteristics of participants who reported no symptoms in their household in the last week and having gone shopping for items other than groceries, toiletries or medicines (non-essentials).

Participants' characteristics	Level	Adherence to lockdown measures			
		Had not gone out shopping for non-essentials; *n* = 1469, *n* (%)	Had gone out shopping for non-essentials; *n* = 476, *n* (%)	Odds ratio (95% CI)	Adjusted odds ratio (95% CI)^a^
Gender	Male	653 (71.4)	262 (28.6)	Reference	Reference
	Female	816 (79.2)	214 (20.8)	0.65 (0.53–0.80)∗∗	0.64 (0.51–0.80)∗∗
Age, years	18–24	57 (75.0)	19 (25.0)	Reference	Reference
	25–34	187 (72.2)	72 (27.8)	1.16 (0.64–2.08)	0.84 (0.43–1.65)
	35–44	267 (76.9)	80 (23.1)	0.90 (0.51–1.60)	0.63 (0.32–1.24)
	45–54	268 (73.8)	95 (26.2)	1.06 (0.60–1.88)	0.74 (0.38–1.44)
	55 and older	690 (76.7)	210 (23.3)	0.91 (0.53–1.57)	0.87 (0.45–1.66)
Have a child in the household	No	1090 (76.3)	338 (23.7)	Reference	Reference
	Yes	363 (73.5)	131 (26.5)	1.16 (0.92–1.47)	1.14 (0.86–1.52)
Clinically extremely vulnerable (self)	No	1333 (75.7)	427 (24.3)	Reference	Reference
	Yes	101 (77.1)	30 (22.9)	0.93 (0.61–1.41)	0.89 (0.57–1.38)
Employment status	Not working	711 (78.7)	192 (21.3)	Reference	Reference
	Working	759 (72.7)	284 (27.3)	1.39 (1.12–1.71)∗	1.61 (1.24–2.09)∗∗
Highest educational or professional qualification	GCSE/vocational/A-level/No formal qualifications	623 (73.8)	224 (26.2)	Reference	Reference
	Degree or higher (Bachelors, Masters, PhD)	810 (77.0)	24 (23.0)	0.84 (0.68–1.04)	0.89 (0.71–1.13)
IMD	More deprived area	631 (74.1)	220 (25.9)	Reference	Reference
	Less deprived area	838 (76.6)	256 (23.4)	0.88 (0.71–1.08)	0.86 (0.68–1.08)
Social grade	ABC1	909 (76.8)	275 (23.2)	Reference	Reference
	C2DE	560 (73.6)	201 (26.4)	1.19 (0.96–1.46)	1.29 (1.01–1.63)∗
Urban/rural	Urban	1110 (75.9)	352 (24.1)	Reference	Reference
	Rural	319 (73.7)	114 (26.3)	1.13 (0.88–1.44)	1.23 (0.94–1.62)
Living alone	Yes	308 (76.6)	94 (23.4)	Reference	Reference
	No	1161 (75.2)	382 (24.8)	1.08 (0.83–1.40)	1.02 (0.70–1.49)
Marital status	Married/civil partnership/living as married	928 (75.3)	305 (24.7)	Reference	Reference
	Separated/divorced/widowed/never married	531 (75.7)	170 (24.3)	0.97 (0.79–1.21)	1.03 (0.75–1.42)
Clinically extremely vulnerable (household member)^b^	No	1041 (75.8)	333 (24.2)	Reference	Reference
	Yes	92 (73.6)	33 (26.4)	1.12 (0.74–1.70)	1.17 (0.74–1.84)
Home includes access to outside space	No	111 (76.0)	35 (24.0)	Reference	Reference
	Yes	1358 (75.5)	441 (24.5)	1.03 (0.69–1.53)	1.09 (0.69–1.70)
Pet ownership	No	813 (75.8)	259 (24.2)	Reference	Reference
	Yes	656 (75.1)	217 (24.9)	1.04 (0.84–1.28)	0.97 (0.77–1.23)

∗*P* ≤ 0.05.

∗∗*P* ≤ 0.001.

a Adjusting for gender, age, having a child in the household, being extremely clinically vulnerable oneself, employment status, highest level of education or professional qualification, indices of multiple deprivation, social grade, living in a rural or urban area, living alone, marital status and region.

b Adjusted analyses for this variable did not control for living alone, as by definition all participants asked this question lived in a household with someone else.

Shopping for non-essentials in the past week was associated with: thinking you have had COVID-19; helping someone outside your household; thinking that you will lose touch with friends or relatives if you follow government guidance; and thinking that following government guidance will negatively impact you financially (see Table 7). Not going out shopping for non-essentials was associated with: having received help from someone outside your household in the last 7 days; reporting that you were self-isolating; increased perceived likelihood of catching and spreading COVID-19; increased worry about COVID-19; increased perceived effectiveness of government advice; increased perceived severity of COVID-19; increased perceived disapproval from friends or family if you do not follow government advice; increased perceived legal consequences of not following government advice; not knowing or being unsure about government measures; and decreased perceived social norms.

**Table 7:** Associations between psychological and situational factors and having gone shopping for items other than groceries, toiletries or medicines (non-essentials) in the past 7 days in participants who reported no symptoms in the household.

Participants' characteristics	Level	Adherence to lockdown measures			
		Had not gone out shopping for non-essentials; *n* = 1469, *n* (%)	Had gone out shopping for non-essentials; *n* = 476, *n* (%)	Odds ratio (95% CI)	Adjusted odds ratio (95% CI)^a^
Had COVID-19	Think have not had COVID-19	1176 (76.8)	356 (23.2)	Reference	Reference
	Think have had COVID-19	104 (67.1)	51 (32.9)	1.62 (1.14–2.31)∗	1.72 (1.17–2.53)∗
Self-isolating	Not self-isolating	1098 (73.6)	393 (26.4)	Reference	Reference
	Self-isolating	371 (81.7)	83 (18.3)	0.63 (0.48–0.81)∗∗	0.61 (0.45–0.83)∗
Understanding of government measures, if no-one in household was symptomatic	Incorrect/unsure	768 (73.0)	284 (27.0)	Reference	Reference
	Correct	701 (78.5)	192 (21.5)	0.74 (0.60–0.91)∗	0.77 (0.61–0.97)∗
Understanding of government measures, if someone in household was symptomatic	Incorrect/unsure	1392 (75.9)	442 (24.1)	Reference	Reference
	Correct	77 (69.4)	34 (30.6)	1.39 (0.92–2.11)	1.27 (0.81–1.99)
Worry about COVID-19	5-point scale, 1 = not at all worried to 5 = extremely worried	N = 1465, M = 3.40, SD = 0.97	N = 473, M = 3.01, SD = 1.00	0.67 (0.60–0.74)∗∗	0.66 (0.59–0.75)∗∗
Perceived social norms	Percentage (range 0–100)	N = 1312, M = 74.19, SD = 15.62	N = 431, M = 70.35, SD = 17.54	0.99 (0.98–0.99)∗∗	0.99 (0.98–0.99)∗∗
Perceptions about impact on mental health	5-point scale, 1 = a lot better to 5 = a lot worse	N = 1452, M = 3.43, SD = 0.87	N = 470, M = 3.43, SD = 0.92	1.00 (0.89–1.13)	1.01 (0.89–1.14)
Perceptions about impact on physical health	5-point scale, 1 = a lot better to 5 = a lot worse	N = 1457, M = 3.15, SD = 0.91	N = 470, M = 3.11, SD = 0.98	0.95 (0.85–1.07)	0.95 (0.84–1.07)
Self-reported general health	5-point scale, 1 = poor to 5 = excellent	N = 1457, M = 3.05, SD = 1.06	N = 473, M = 3.06, SD = 1.01	1.01 (0.92–1.12)	1.05 (0.94–1.17)
Helped someone outside household	No	1138 (77.5)	331 (22.5)	Reference	Reference
	Yes	321 (69.9)	138 (30.1)	1.48 (1.17–1.87)∗∗	1.56 (1.21–2.01)∗∗
Received help from someone outside household	No	1234 (74.1)	431 (25.9)	Reference	Reference
	Yes	225 (85.6)	38 (14.4)	0.48 (0.34–0.69)∗∗	0.53 (0.36–0.78)∗∗
If I completely follow the government's advice, I will lose touch with my friends and relatives	5-point scale, 1 = strongly disagree to 5 = strongly agree	N = 1456, M = 1.94, SD = 1.04	N = 469, M = 2.25, SD = 1.18	1.28 (1.17–1.40)∗∗	1.30 (1.17–1.44)∗∗
My friends or family will disapprove, if I don't follow the government's advice	5-point scale, 1 = strongly disagree to 5 = strongly agree	N = 1428, M = 4.11, SD = 0.93	N = 466, M = 3.8, SD = 1.05	0.73 (0.66–0.81)∗∗	0.73 (0.65–0.81)∗∗
If I don't follow the government's advice, I could get in trouble with the police	5-point scale, 1 = strongly disagree to 5 = strongly agree	N = 1448, M = 3.98, SD = 0.85	N = 468, M = 3.77, SD = 0.97	0.77 (0.69–0.87)∗∗	0.78 (0.69–0.88)∗∗
If I follow the government's advice, it will help save lives	5-point scale, 1 = strongly disagree to 5 = strongly agree	N = 1458, M = 4.54, SD = 0.72	N = 472, M = 4.26, SD = 0.95	0.67 (0.59–0.75)∗∗	0.66 (0.58–0.75)∗∗
If I follow the government's advice, it will help protect the NHS	5-point scale, 1 = strongly disagree to 5 = strongly agree	N = 1458, M = 4.56, SD = 0.74	N = 471, M = 4.32, SD = 0.90	0.71 (0.62–0.80)∗∗	0.71 (0.62–0.81)∗∗
If I catch coronavirus, I may become very ill	5-point scale, 1 = strongly disagree to 5 = strongly agree	N = 1448, M = 4.43, SD = 0.82	N = 469, M = 4.18, SD = 0.94	0.73 (0.65–0.82)∗∗	0.72 (0.63–0.81)∗∗
If I catch coronavirus, it will have a severe impact on my family's well-being	5-point scale, 1 = strongly disagree to 5 = strongly agree	N = 1431, M = 4.12, SD = 1.01	N = 463, M = 3.86, SD = 1.10	0.80 (0.72–0.88)∗∗	0.79 (0.71–0.88)∗∗
If I leave home and meet other people, I could pass coronavirus to someone else	5-point scale, 1 = strongly disagree to 5 = strongly agree	N = 1453, M = 4.44, SD = 0.81	N = 471, M = 4.14, SD = 0.97	0.69 (0.62–0.78)∗∗	0.66 (0.58–0.75)∗∗
If I leave home and meet other people, I could catch coronavirus	5-point scale, 1 = strongly disagree to 5 = strongly agree	N = 1459, M = 4.45, SD = 0.74	N = 470, M = 4.14, SD = 0.88	0.64 (0.56–0.72)∗∗	0.59 (0.52–0.68)∗∗
If I follow the government's advice, it will have a negative impact on how much money I have	5-point scale, 1 = strongly disagree to 5 = strongly agree	N = 1426, M = 2.47, SD = 1.20	N = 467, M = 2.64, SD = 1.24	1.12 (1.03–1.22)∗	1.13 (1.03–1.24)∗
Because of the current lockdown, there is more conflict between people that I live with	5-point scale, 1 = strongly disagree to 5 = strongly agree	N = 1406, M = 2.08, SD = 1.15	N = 453, M = 2.23, SD = 1.16	1.11 (1.01–1.22)∗	1.07 (0.97–1.19)
If I follow the government's advice, I will not be able to carry out important religious activities	5-point scale, 1 = strongly disagree to 5 = strongly agree	N = 1294, M = 2.59, SD = 1.35	N = 425, M = 2.70, SD = 1.33	1.06 (0.98–1.15)	1.08 (0.99–1.18)
I am enjoying spending more time at home during the lockdown	5-point scale, 1 = strongly disagree to 5 = strongly agree	N = 1458, M = 3.29, SD = 1.20	N = 473, M = 3.21, SD = 1.21	0.95 (0.87–1.03)	0.94 (0.86–1.03)
Because of coronavirus, I feel a sense of community with other people in my neighbourhood	5-point scale, 1 = strongly disagree to 5 = strongly agree	N = 1455, M = 3.36, SD = 1.07	N = 470, M = 3.30, SD = 1.06	0.94 (0.86–1.04)	1.00 (0.90–1.11)

∗*P* ≤ 0.05.

∗∗*P* ≤ 0.001.

a Adjusting for gender, age, having a child in the household, being extremely clinically vulnerable oneself, employment status, highest level of education or professional qualification, indices of multiple deprivation, social grade, living in a rural or urban area, living alone, marital status and region.

#### Power

For analyses where no symptoms were present in the household, we achieved 100% power to detect small effect sizes in logistic regression analyses (OR = 1.68,^9^ α = .05, sample size *n* = 1945, probability of having gone out shopping for items other than groceries, toiletries or medicines = 0.25, one-tailed and two-tailed logistic regression). We achieved 94% power to detect small effect sizes in linear regression analyses (*f*^2^ = 0.02,^10^ α = .05, sample size *n* = 1945, number of tested predictors = 39, total number of predictors = 39).

## Discussion

To the best of our knowledge, this is the first comprehensive study to investigate factors associated with self-isolation and behaviour during lockdown in the UK. Almost 10% of participants reported that either they or a household member had symptoms of COVID-19 (a cough or high temperature/fever) in the last week. Prevalence estimates by the UK Office for National Statistics indicate that at the time of data collection, 0.27% of the community population had COVID-19.^11^ Government regulations required all those with symptoms, or with symptoms in their household, to self-isolate. Our results suggest that adherence to this is poor. Three-quarters of those with symptoms in their household reported leaving their home in the past 24 h. We found no difference in out-of-home behaviour by presence of symptoms in the household. The UK will shortly enter a new phase of the pandemic, in which extensive testing, contact tracing and isolation will be required to keep the spread of COVID-19 in check.^12^ For this to succeed, adherence must be improved. There is some evidence that institution-based isolation is more effective compared to home-based isolation, in part because this is less reliant on personal adherence to guidelines.^13^ Some countries have used large-scale, temporary shelter hospitals, which are primarily for patients with mild and moderate symptoms of COVID-19. Shelter hospitals allow patients to isolate effectively from their family and community; be triaged, reducing pressure on other health care services; provide basic medical care; frequent monitoring and rapid referral if a patients’ symptoms worsen; and provide living and social support.^14^

Our findings highlight several risk factors for poor adherence. Notably men were more likely to report having been out in the last 24 h if they or someone in their household was symptomatic, having gone out more times in the last week and shopping for non-essentials. Lower adherence among men was also noted in the UK during the 2009/10 H1N1 influenza pandemic.^15^ Communication campaigns that specifically target men may therefore have merit.

Adherence with self-isolation was associated with increased worry about COVID-19 and increased perceived likelihood of catching COVID-19. As incidence declines, it is possible that worry will also decline, reducing adherence further. Although it may be tempting to use fear-based messaging to combat this, this may influence other behaviours that the government may wish to encourage, such as return to work.^16^

Adherence was also associated with having received help from someone outside your household. This makes intuitive sense—having someone else to run errands should reduce the need for you to leave home. Much has been made recently of the remarkable altruism of 750,000 people who signed-up to volunteer for the National Health Service, and the lack of jobs for them to do.^17^ Allowing those in self-isolation to submit requests for help may be a pragmatic way to improve adherence.

Adherence to lockdown measures among those not reporting symptoms in their household was better, but still not perfect, with 75% reporting not going out to shop for non-essential items. Percentages reporting not meeting up with friends or family from outside one's household and not having visitors to the home were higher (94% and 95%, respectively). Adherence was lower in men and those who reported working. It is plausible that workers may be more likely to be out and about for work and while out, go shopping for non-essentials. Those working may also be more financially able to shop for non-essential items. Although perceiving greater negative financial consequences of government measures was associated with non-adherence to lockdown measures, there was no longer evidence for an association after correcting for multiple adjustments. This is different from research finding decreased intention to adhere to quarantine measures in Israel.^18^ Adherence to lockdown measures was also associated with higher threat appraisals and positive appraisals of the coping response. These findings mirror research in other countries.^19^, ^20^, ^21^ Non-adherence was associated with decreased perceived social norms,^19^^,^^22^ lower perceived social pressure to adhere to measures and decreased knowledge of measures.^5^ These findings suggest that improvement in adherence to lockdown measures is likely to be achieved by emphasising these are actions that most people are taking, that are having a positive impact, and that others around you want you to do.

This study has several limitations. First, despite using quota sampling, we cannot be sure that survey respondents are representative of the general population.^23^^,^^24^ Second, all data were self-reported and may have been susceptible to social desirability bias.^25^ However, preliminary data indicate that self-reported physical distancing is associated with real-world behaviour.^26^ Third, we did not ask participants if they came into close contact with anyone from another household while they were out and about. Clearly, non-adherence does not always increase the risk of disease transmission. Fourth, we used a cumulative measure of ‘outings’ for our outcome measure. It is possible that participants may have shopped for essentials and non-essentials in the same trip, which might be double-counted in our questionnaire. Fifth, the cross-sectional nature of data collection means we are unable to draw causal inferences. Sixth, although the total sample size was large, a small percentage of the population reported that they or someone in their household had experienced symptoms of COVID-19 in the last week. Thus, analyses investigating adherence to self-isolation were based on smaller sample sizes, resulting in decreased power and wider confidence intervals.

Overall, our data suggest that self-reported adherence to self-isolation measures was poor. This has important implications for policies that attempt to prevent the spread of COVID-19 through self-isolation, such as contact tracing. Psychological factors including perceived effectiveness of lockdown measures, should be emphasised in communications. Effective use of volunteer programmes and help within the neighbourhood or community may also improve adherence.

## Author statements

### Funding sources

LS, RA and GJR are supported by the National Institute for Health Research Health Protection Research Unit (NIHR HPRU) in Emergency Preparedness and Response, a partnership between Public Health England, King's College London and the University of East Anglia. RA, HL, IO and CR are supported by the NIHR HPRU in Behavioural Science and Evaluation, a partnership between Public Health England and the University of Bristol. CR is also supported by the NIHR HPRU in Emerging and Zoonotic Infections and NIHR HPRU in Gastrointestinal Infections. The views expressed are those of the authors and not necessarily those of the NIHR, Public Health England or the Department of Health and Social Care. The 10.13039/501100000272NIHR HPRU Emergency Preparedness and Response funded the study. An 10.13039/501100000265MRC award under the MRC COVID-19 Rapid Response call (grant number MC_PC_19071) funded RA, HL, IO, CR, LY and GJR's time.

### Statement of ethical approval

Ethical approval for this study was granted by the King's College London Research Ethics Committee (reference: LRS-19/20-18687).

### Data sharing statement

Anonymised data will be made available on reasonable request.

### Author contribution statement

The study was conceptualised by RA, HL, IO, CR, LY and GJR. LS completed all analyses, using data from YouGov Plc. All authors contributed to, and approved, the final manuscript. For any enquiries about the data in this report please contact King's College London.
